# Self-assembled monolayers of alkanethiolates on surface chemistry groups in osteosarcoma cells

**DOI:** 10.3892/mmr.2014.2876

**Published:** 2014-11-06

**Authors:** YING-HU DENG, LI-HUA LI, JIN HE, MEI LI, YU ZHANG, XIU-MEI WANG, FU-ZHAI CUI, HONG XIA

**Affiliations:** 1 Department of Orthopaedics Surgery, The First Clinical Medical College, Southern Medical University, Guangzhou, Guangdong 510515, P.R. China; 2 Department of Orthopaedics, Tongling People’s Hospital, Tongling, Anhui 244000, P.R. China; 3 Department of Spinal Surgery, Hospital of Orthopedics, Guangzhou General Hospital of Guangzhou Military Command, Guangzhou, Guangdong 510010, P.R. China; 4 Department of Materials Science and Engineering, Institute of Regenerative and Biomimetic Materials, Tsinghua University, Beijing 100084, P.R. China

**Keywords:** osteosarcoma, functional groups, self-assembled monolayers

## Abstract

Cell biomedical behavior is influenced by a number of factors, and the extracellular matrix (ECM) of the cellular microenvironment affects certain cancer cells. In the current study, U-2OS cells were cultured on gold surfaces modified with different terminal chemical groups [methyl (-CH_3_), amino (-NH_2_), hydroxyl (-OH) and carboxyl (-COOH)]. The results revealed that different chemical surfaces convey different behaviors. The density of the different functional surfaces was confirmed by atomic force microscopy. Cell morphology, proliferation rate and cell cycle were investigated using scanning electron microscopy, cell counting and flow cytometry. In conclusion, the type of chemical group on a biomaterial is an important property for the growth of osteosarcoma cells; -NH_2_ and -COOH surfaces sustained visible cell adhesion and promoted cell growth.

## Introduction

The self-assembled monolayers (SAMs) technique comprising alkanethiolates on gold is well established (1,2). SAMs serve as a good model of the extracellular matrix (ECM) with different terminal functional groups (3). These substrate-dependent differences in protein adsorption have profound effects on cellular activities, including cell adhesion, proliferation, migration and differentiation (4–7). The SAMs on the gold surface provide chemical functional group surfaces for cell behaviors. A number of studies have focused on cell behavior in response to different chemical groups using the SAM technique, including osteoblastic cells (8), murine fibroblasts (9) and mesenchymal stem cells (10). SAMs greatly affect cancer cells, including breast cancer (11) and hepatoma (12). Osteosarcoma is one of the most common types of primary malignant skeletal tumor in children and adolescents (13). It occurs primarily around the distal femur, proximal tibia and humerus. Other significant locations include the proximal femur, pelvis, skull and jaw (14). The aim of this study was to investigate the associations between chemical functional groups and the behaviors of U-2OS human osteosarcoma cells, including the phenotype, adhesion, proliferation and apoptosis. The effects of chemical groups on U-2OS cells will aid in improving the understanding of the regulatory mechanisms of biomaterials on osteosarcoma cells and may provide a novel way to use biomaterials to treat and prevent the recurrence of osteosarcoma.

## Materials and methods

### Preparation of model surfaces on glass coverslips

SAMs were prepared using a protocol modified from our previous study (15). Briefly, the Au surfaces (40 nm) were deposited on glass coverslips following a Ti layer (40 nm), using an ANELVAL-400EK electron beam evaporator (Canon Anelva Corp., Kanagawa, Japan). The silicon wafers coated with Au (Zhongding Ltd., Yangzhou, China) were washed with triple-distilled water in an ultrasonic bath for 10 min, and immersed in 1 Mm 1-undecanethiol (-CH_3_; Sigma, St. Louis, MO, USA), 11-amino-1-undecanethiol (-NH_2_; Sigma), 11-hydroxy-1-undecanethiol (-OH; Sigma), 12-mercaptododecanoic acid (-COOH; Sigma) for 12 h (16). Following the self-assembly process, the substrates were washed with triple-distilled water and dried with nitrogen.

### Characterization of substrates

Different groups with -CH_3_, -NH_2_, -OH or -COOH surfaces were characterized by contact angle measurements. Ambient air-water substrate contact angle measurements (4 ml ultra-pure H_2_O) were performed using a OCA20 contact angle system (Dataphysics, Filderstadt, Germany) fitted with a digital camera, and analyzed using in-house image analysis software.

### Cell culture

The human osteosarcoma U-2OS cell line was obtained from the American Type Culture Collection (Manassas, VA, USA). U-2OS cells were cultured in RPMI-1640 (Hyclone, Logan, UT, USA) containing 100 U/ml penicillin (Hyclone) and 100 mg/ml streptomycin (Hyclone), supplemented with 10% fetal bovine serum (FBS; Invitrogen Life Technologies, Carlsbad, CA, USA) at 37°C in 5% CO_2_ and 95% air. U-2OS cells were maintained in 10% FBS RPMI-1640 and passaged every 2 days.

### Cell adhesion

U-2OS cells (5×10^3^ cells/ml) were seeded onto the different surfaces of the SAMs. Following a 3-, 6- and 9-h culture, cell adhesion analyses were performed under an inverted microscope (HBOSO/AC Hg lamp, Oberkochen, Germany; BX-51 microscope, Leica, Germany). Further adhesion analysis of the cells cultured for 6 h was performed by scanning electron microscopy (SEM; Hitachi, H50, Hitachi, Ltd., Tokyo, Japan).

The U-2OS cells attached to the surface of the SAMs could be visualized clearly under the inverted phase contrast microscope, so three different time points were selected to for investigation. The cell morphology was examined, and the attached cell numbers were counted in five random fields.

The morphology of the cells on the sample surfaces was further examined by SEM. Following cultivation for 6 h, the samples were removed from the culture plates and fixed with 3% glutaraldehyde (Sigma-Aldrich, St. Louis, MO, USA) for 6 h. Following this, they were washed three times with PBS (10 min each time) and dehydrated sequentially in a series of ethanol (50, 70, 95 and 100%; each concentration twice for 10 min each time) and air dried in a fume hood. Following sputter-coating with gold, samples were examined by SEM, and the adhesion and spreading of the cells in the different groups with modified substrates were observed by SEM (Hitachi, H50).

### Cell viability analysis

Cell viability was analyzed by live/dead cell staining, MTT and lactate dehydrogenase (LDH) measurement. For viability staining studies, cells were seeded in 24-well plates at a concentration of 1×10^4^ cells/ml at 37°C with 5% CO_2_. Cells were grown to confluence. The gold substrates were added to the 24-well plates prior to seeding and incubation for 24 h. At the end of the incubation period, the media was removed and the adherent cells were subjected to live/dead staining following the manufacturer’s instructions (calcein AM; 17783-1MG; Sigma-Aldrich). The viability of the different chemical groups on the cells was conducted using the viability/cytotoxicity staining method following the manufacturer’s instructions (Sigma-Aldrich). Briefly, 1 μM calcein AM (17783-1MG Sigma-Aldrich) and 2 μM ethidium homodimer-1 solutions (EthD-1, AnaSpec 83208, 1 mg, AnaSpec, Fremont, CA, USA) were prepared in phosphate-buffered saline. Following removal of the culture medium, the cells were washed once in phosphate buffered saline (PBS; Sigma-Aldrich), 100 μl of 1 μM calcien AM and 2 μM ethidium homodimer-1 solution were added and the cells were incubated for 30 min. Cell images were captured using a fluorescence microscope (Olympus, BX51, Olympus Corporation, Tokyo, Japan); the dyed red and green cells were counted in five random fields.

### Cell proliferation

The cell proliferative ability was tested using an MTT assay (n=3 donors). The optical density (O.D.) value was tested 2, 4 and 6 days post-culture. At each point, 30 μl of MTT (5 mg/ml) solution was added to 300 μl medium, and the cells incubated at 37°C for 4 h. The culture medium was removed and the cells washed in PBS three times. The formazan reaction products were dissolved in dimethylsulfoxide (DMSO; Sigma-Aldrich) for 10 min. The O.D. of the formazan solution was measured on an ELISA plate reader (Thermo, Multiskan G; Thermo Fisher Scientific, Waltham, MA, USA) at 490 nm.

LDH is a cytoplasmic enzyme, often associated with cell membrane damage and cell death (17). The LDH activity was measured spectrophotometrically by assaying reduced nicotinamide adenine dinucleotide oxidation at a wavelength of 340 nm during the LDH-catalyzed reduction of pyruvate to lactate. Briefly, cells were cultured with gold substrates in 24-well plates for 24 h. The supernatant was then removed and centrifuged to eliminate the non-adherent cell debris. Adherent cells were lysed with 0.5% Triton X-100. Samples of each chemical group were then analyzed spectroscopically.

### Cell apoptosis and necrosis

Apoptosis is a form of programmed cell death that occurs through the activation of intrinsic cell suicide machinery (18). To analyze changes in nuclear morphology, U-2OS cells were measured using Guava Nexin Reagent (Millipore, Billerica, MA, USA). Following culture with different chemical groups, the cells in contact with the substrates were washed with PBS and centrifuged at 200 × g for 5 min. The cell pellets were suspended in 100 μl RPMI-1640 medium supplemented with 1% FBS, then incubated with 100 μl of Annexin V-PE and 7-AAD labeling solution for 20 min at room temperature. Cells were analyzed on a Guava EasyCyte 5HT flow cytometer (Millipore) using a 488-nm excitation and a 575-nm bandpass filter for PE detection, and a 546-nm excitation and a 647-nm filter for 7-AAD detection. The data were analyzed using the Guava Nexin Software v2.2.2 (Millipore).

### Statistical analysis

All statistics were performed with the Origin Pro 8.0 software package (OriginLab, Guangzhou, China). The data were analyzed by one way analysis of variance using SPSS 13.0 (SPSS, Inc., Chicago, IL, USA). P<0.05 was considered to indicate a statistically significant difference.

## Results

### Physicochemical characterization of different model surfaces

**Figure 1:**
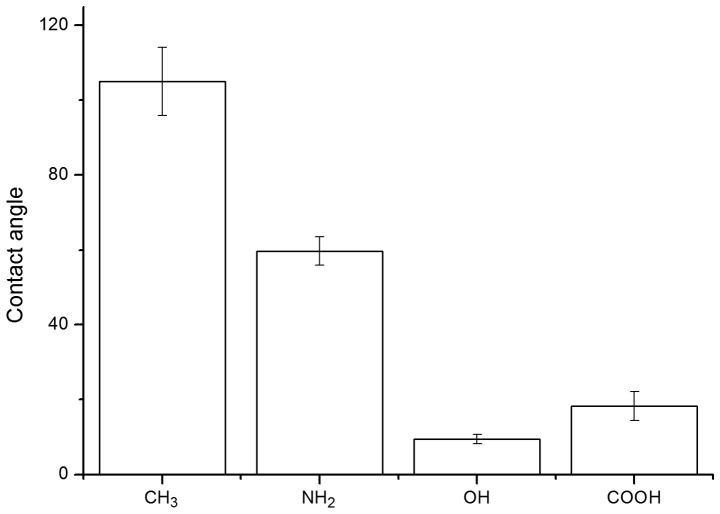
Self-assembled monolayer surfaces characterized by water contact angles. Results are presented as the mean ± standard deviation of six independent experiments, each performed in triplicate.

The results of contact angle measurements are shown in Fig. 1. Among these test surfaces, the -OH surface was the most hydrophilic, with a contact angle of 9.5°±1.2°. The -COOH surface had a slightly higher contact angle value of 18.3°±3.9°. The contact angle of the -NH_2_ surface was 59.7°±3.8°, although -NH_2_ was still classified as hydrophilic. Due to its non-polar nature, the -CH3 surface had the highest contact angle of 105.0°±9.1°, which was the most hydrophobic surface in these tests.

### The adhesion of U-2OS cells

The adhesion of U-2OS cells to the chemical group-modified substrates was investigated using microscopy and SEM.

**Figure 2:**
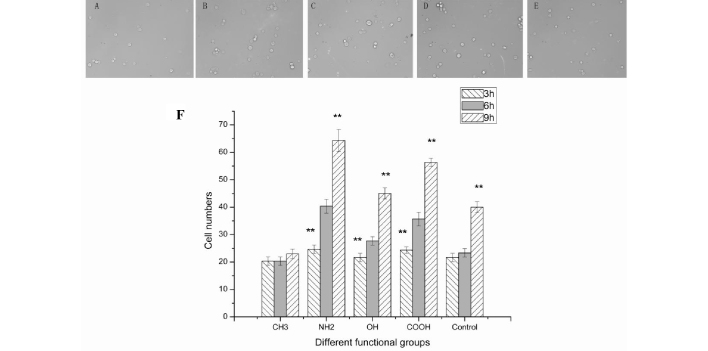
The U-2OS cells cultured on surfaces with different chemical functional groups for 6 h (magnification, ×200). (A) -CH_3_ surface; (B) -NH_2_ surface; (C) -OH surface; (D) -COOH surface. (E) Control group. (F) Cell numbers on different functional groups in 3, 6 and 9 h. ^*^P<0.05; ^**^P<0.01 compared with the same substrates at 6 h.

Cell adhesion numbers on the different chemical surfaces after 3-, 6- and 9-h incubations are shown in Fig. 2. The adherent cell number of -CH_3_ group remained at the initial level. In contrast, the cell number on the -OH, -NH_2_ and -COOH surfaces was dramatically increased compared with that on -CH_3_ surface. The cell number on the -NH_2_ surface was higher than the other three groups during the incubation period. For the -NH_2_, -OH, -COOH groups, the number of cells increased accordingly. However, there were no clear differences between the -NH_2_ and -COOH groups. In addition, the adhesion number of the U-2OS cells followed the trend: -CH_3_ << -OH < -COOH ≈ -NH_2_.

**Figure 3:**
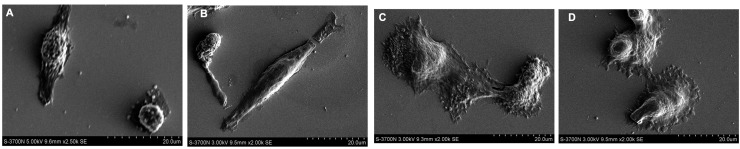
The scanning electron microscopy images of U-2OS cells cultured on surfaces with different chemical functional groups for 6 h. (A) -CH_3_; (B) -NH_2_; (C) -OH; and (D) -COOH surfaces. Scale bar, 20 μm.

In order to further prove the different morphology changes, SEM (Fig. 3) was used. The results indicated that U-2OS cells cultured on the -OH and -COOH functional groups exhibit polygonal and oval morphology, those cultured on NH_2_ showed a spindle shape and cells cultured on -CH_3_ group were smaller and in a spherical shape, which was in accordance with the phase microscope results.

### Cell viability

**Figure 4:**
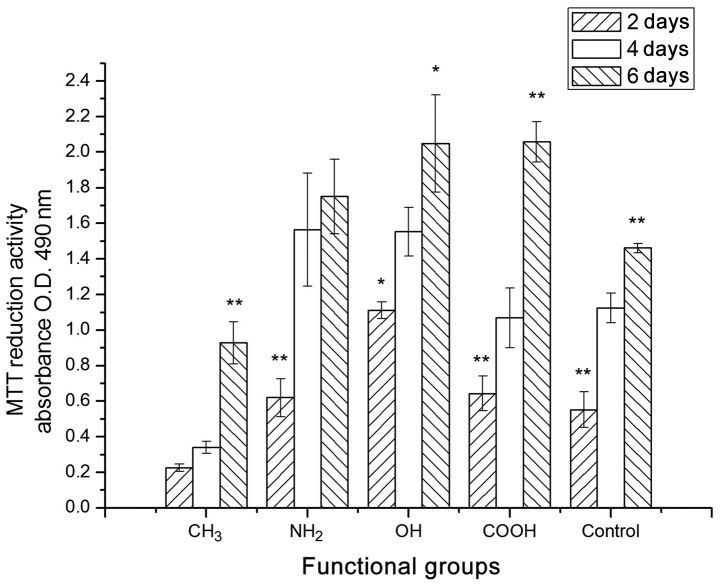
MTT activity assay of U-2OS cultured on different chemical group-modified substrates at days 2, 4 and 6. Results represent the mean ± standard deviation of three independent cultures and determinations. ^*^P<0.05,^**^P<0.01 compared with the same substrates at 4 days, as determined by one way analysis of variance. O.D., optical density.

The MTT results for the U-2OS cells cultured on the different chemical group-modified surfaces on days 2, 4 and 6 are shown in Fig. 4. The U-2OS cells on the different surfaces were uniformly seeded at the beginning of the experiment. From day 2 to day 6, there was a significant increase in the proliferation rate of U-2OS cells on the surfaces with the -CH_3_, -NH_2_, -OH and -COOH groups. However, the cells on the -CH_3_ surface had a low O.D. level compared with that of the other functional groups from Day 2 to Day 6. The cells in the control showed a similar moderate proliferation level to the -OH groups. It appears that the -COOH and -NH_2_ groups had a promoting effect on the proliferation of U-2OS cells in a longer culture period. However, the -CH_3_ group had a negative effect on U-2OS proliferation. Furthermore, the proliferation capacity of U-2OS on the different surfaces followed the trend: -COOH > -OH ≥ -NH_2_ >> -CH_3_.

**Figure 5:**
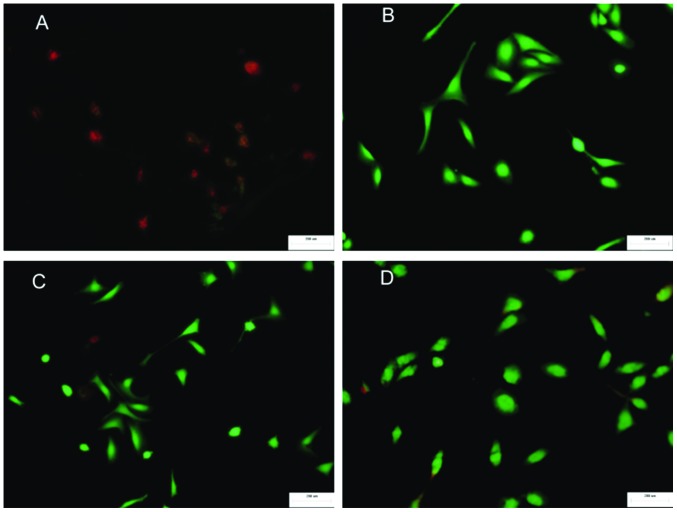
Fluorescence micrographs of live/dead dye-stained U-2OS cells cultured on different chemical group-modified substrates at 24 h. (A) -CH_3_; (B) -NH_2_; (C) -OH; and (D) -COOH surfaces. Scale bar, 200 μm. Green cells are viable cells; red cells are non-viable cells.

The effects of different chemical groups on cell survival were also evaluated using live/dead viability and cytotoxicity staining (Fig. 5). It was determined that there were few cells on the -CH_3_ surface, which primarily consisted of dead cells. By contrast, on the -COOH, -NH_2_ and -OH surfaces, there were a greater number of viable cells in a markedly larger contact area. The cells that were exposed to a larger area and the morphology were consistent with the SEM results. The survival rates of cells on different surfaces followed the trend: -NH_2_ > -OH ≥ -COOH >> -CH_3_. These results are supported previous studies that found that the -NH_2_ functional group diminishes cell toxicity while -CH_3_ exhibits cell toxicity (11,12).

### LDH

**Figure 6:**
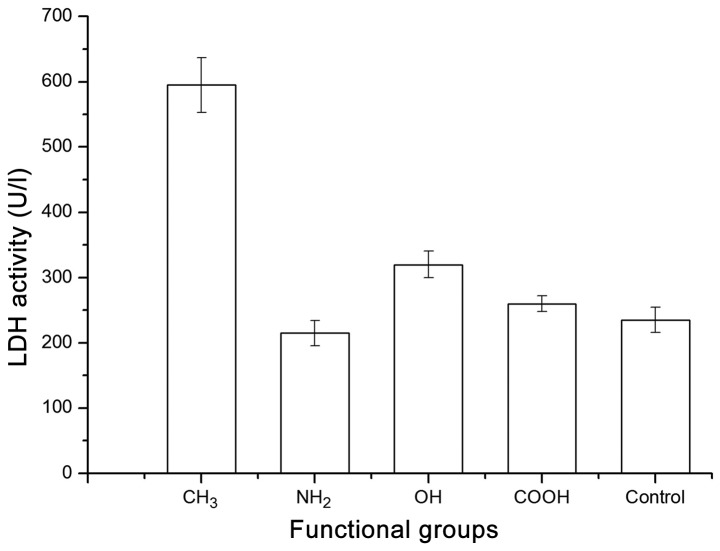
Lactate dehydrogenase (LDH) activity of U-2OS cells cultured on different chemical group-modified substrates at 24 h. Results are presented as the mean ± standard deviation of three independent experiments, each performed in triplicate.

The present results indicated that the -CH_3_ group may interrupt the continuity of the cell membrane and subsequently lead to membrane breakdown and cytoplasm leakage. To test this assumption, the release of the cytoplasmic enzyme LDH by adherent cells was measured on chemical group-modified substrates. Assays of the LDH activities revealed the following trend: -CH_3_ > -OH ≥ -COOH > -NH_2_, which were in agreement with the MTT results, demonstrating the association between cell toxicity and the release of LDH enzymes (Fig. 6).

### Cell apoptosis and necrosis

**Figure 7:**
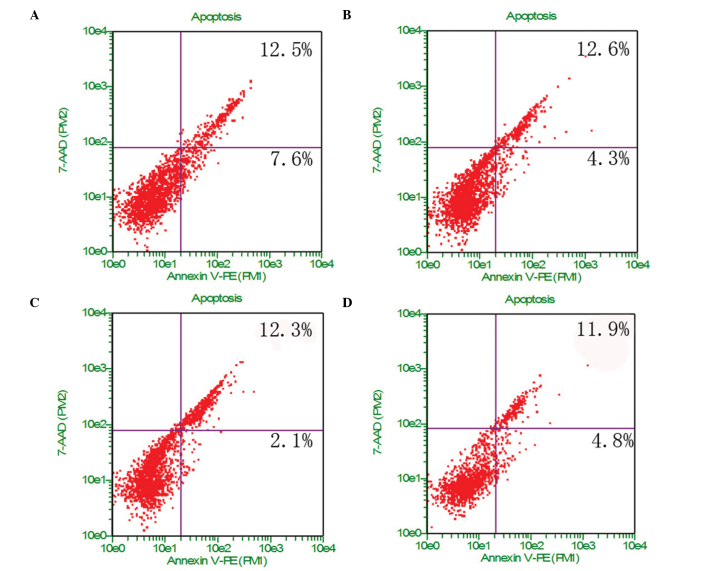
Apoptosis and necrosis of U-2OS cultured on surfaces with different chemical functional groups after 24 h of culture. (A) -CH_3_; (B) -NH_2_; (C) -OH; and (D) -COOH groups.

U-2OS cells in different chemical groups were analyzed for apoptosis using Annexin V-PE and 7-AAD. Annexin V-PE was used to detect phosphatidylserine (PS) on the external membrane of apoptotic cells. 7-AAD, the cell impermeant dye, is used as an indicator of cell membrane structural integrity. As shown in Fig. 7, the -CH_3_ group caused ~7.6% apoptosis and ~12.5% necrosis, whereas the -NH_2_ group caused ~4.3% apoptosis and ~12.6% necrosis. The apoptosis rate showed the following trend: -CH_3_ > -COOH > -NH_2_ ≥ -OH. These results indicate that the -NH_2_ surface exhibits improved cell biocompatibility, and the -CH_3_ group may cause death by apoptosis and early apoptosis.

## Discussion

In the current study, -CH_3_, -OH, -NH_2_ and -COOH were selected to construct SAMs on bare gold surfaces, and these four types of chemical groups were used to further study their effects on U-2OS cells. The functional groups showed significant effects on cell morphology, adhesion, proliferation and apoptosis. Adhesion and spreading of cells on biomaterials via the ECM are integrin-mediated processes, and cells use different adhesion mechanisms for the exploration of the material’s surface. Surface chemistry has been reported to affect cell interactions in a number of studies (9,12,13,19), particularly cell morphology and adhesion. In this research, U-2OS cells adhered and spread well on -OH, -NH_2_ and -COOH terminal groups, which is in accordance with previous studies (9,12,13). However, the cells cultured on the -CH_3_ surface occupied a small area and were spherical in shape, typical non-proliferating cell characters, which is likely to exhibit cell toxicity and promote cell apoptosis (15,16). Cells cultured on -OH and -COOH terminal groups had a polygon shape, while the cells on -NH_2_ surface exhibited spindle and polygon morphologies. The cells cultured on these functional groups increased from 3 to 24 h, and the density of the cells on -OH and -NH_2_ surfaces was similar (Fig. 2D). Cellular activity is primarily dependent on the surface of the materials, including roughness (20), chemical composition (21) and hydrophilicity (22). The -OH group is hydrophilic, while -CH_3_ is hydrophobic when the charge is neutral. This difference is the leading cause of different cell adhesion and spreading.

Cell adhesion and morphology are tightly linked with cell viability (19). Cells cultured on -COOH and -NH_2_ markedly promote cell proliferation, while the -CH_3_ functional group inhibits cell proliferation, which is in accordance with previous studies (12,13). The cell proliferation was inversely correlated with cell toxicity (LDH and viability/cytotoxicity staining). The integrity of the cell membrane is crucial to maintain its viability, which means that the cells will undergo necrosis if the membrane is broken (23). LDH release has been chosen to determine the ratio of live to dead cells. In the current study, the same method was used to check the toxicity of the chemical functional group. It was determined that -CH_3_ causes the largest amount of LDH release, indicating that -CH_3_ may lead to the majority of cells to apoptosis and necrosis.

Osteosarcoma is a typical malignant tumor with uncontrolled growth and metastasis. Apoptosis is a genetically programmed cell death which occurs via the activation of intrinsi cell suicide machinery (24). Cell death in a tumor is commonly attributed to the induction of apoptosis. The results of the present study showed that the -CH_3_-cultured cells exhibited early apoptosis, reflected in a shift from green to red cells in cytotoxicity staining. Apoptosis assays in -CH_3_ functional groups are in agreement with cell proliferation and biological behavior. The results revealed that U-2OS cells showed a moderate proliferation rate and little toxicity when cultured on -NH_2_ terminal groups, while a strong proliferation rate when cultured on -COOH group. Yan *et al* (11) showed that MCF-7 cells cultured on -NH_2_ and -COOH surfaces had the best biocompatibility, -OH had the weakest viability and -CH_3_ did not affect cell viability and migration, which was markedly different from the present results. This difference suggests that -CH_3_ and -OH functional groups have distinct roles in different cells.

In the present study, a lower ratio of cell apoptosis existed in all of the cells cultured on different chemical functional groups, but the greatest difference existed between -CH_3_ and the other groups. The -CH_3_ group inhibits the proliferation of U-2OS cells and promotes cell apoptosis, and it may give means to design novel therapeutic agents or biomaterials to treat or prevent the recurrence of osteosarcoma.

In conclusion, the results of this study have shown that the type of chemical group is an important property of biomaterials for the growth of osteosarcoma. -NH_2_ and -COOH surfaces sustained visible cell adhesion and promoted cell growth. Cells cultured on -OH surfaces exhibited similar effects on proliferation but an increased ability to promote apoptosis and death. In contrast, -CH_3_ surfaces showed anticancer effects, inhibiting cell growth, causing poor cell adhesion and increased levels of apoptosis and necrosis.
